# Does pain intensity after total knee arthroplasty depend on somatosensory functioning in knee osteoarthritis patients? A prospective cohort study

**DOI:** 10.1007/s10067-024-06976-7

**Published:** 2024-04-26

**Authors:** Sophie Vervullens, Lotte Meert, Rob J. E. M. Smeets, Jonas Verbrugghe, Peter Verdonk, Mira Meeus

**Affiliations:** 1 Research Group MOVANT, Department of Rehabilitation Sciences and Physiotherapy (REVAKI), University of Antwerp, Wilrijk, Belgium; 2 Research School CAPHRI, Department of Rehabilitation Medicine, Maastricht University, Universiteitssingel 40, 6229 ER Maastricht, The Netherlands; 3 Pain in Motion International Research Group (PiM), , http://www.paininmotion.be; 4 REVAL-Rehabilitation Research Center, Faculty of Rehabilitation Sciences, Hasselt University, Hasselt, Belgium; 5 ORTHOCA, Antwerp, Belgium and ASTARC Department, Antwerp University, Antwerp, Belgium; 6 CIR Clinics in Revalidatie, Location Eindhoven, Maastricht, The Netherlands

**Keywords:** Central sensitization, Chronic postoperative pain, Knee osteoarthritis, Somatosensory functioning, Total knee arthroplasty

## Abstract

The objective of this study is to determine whether the change in pain intensity over time differs between somatosensory functioning evolution profiles in knee osteoarthritis (KOA) patients undergoing total knee arthroplasty (TKA). This longitudinal prospective cohort study, conducted between March 2018 and July 2023, included KOA patients undergoing TKA in four hospitals in Belgium and the Netherlands. The evolution of the Knee Injury and Osteoarthritis Outcome Score (KOOS) subscale pain over time (baseline, 3 months, and 1 year post-TKA scores) was the outcome variable. The evolution scores of quantitative sensory testing (QST) and Central Sensitization Inventory (CSI) over time (baseline and 1 year post-TKA scores) were used to make subgroups. Participants were divided into separate normal, recovered, and persistent disturbed somatosensory subgroups based on the CSI, local and widespread pressure pain threshold [PPT] and heat allodynia, temporal summation [TS], and conditioned pain modulation [CPM]. Linear mixed model analyses were performed. Two hundred twenty-three participants were included. The persistent disturbed somatosensory functioning group had less pronounced pain improvement (based on CSI and local heat allodynia) and worse pain scores 1 year post-TKA (based on CSI, local PPT and heat allodynia, and TS) compared to the normal somatosensory functioning group. This persistent group also had worse pain scores 1 year post-TKA compared to the recovered group (based on CSI). The study suggests the presence of a “centrally driven central sensitization” subgroup in KOA patients awaiting TKA in four of seven grouping variables, comprising their less pain improvement or worse pain score after TKA. Future research should validate these findings further. The protocol is registered at clinicaltrials.gov (NCT05380648).

**Supplementary Information:**

The online version contains supplementary material available at 10.1007/s10067-024-06976-7.

**Table:** 

**Key points** • *A normal, recovered, and persistent disturbed somatosensory functioning group in knee osteoarthritis patients undergoing total knee arthroplasty is proposed based on quantitative sensory testing and the Central Sensitization Inventory.* • *The persistent disturbed somatosensory functioning group classified according to the Central Sensitization Inventory had no pain improvement 1 year after total knee arthroplasty.* • *The persistent disturbed somatosensory functioning group classified according to the Central Sensitization Inventory is a possible “centrally driven disturbed somatosensory functioning” group.*	

## Introduction

Knee osteoarthritis (KOA) is the third most prevalent musculoskeletal disorder in the world [1], causing substantial chronic pain and disability [2]. When conservative treatments are ineffective, and patients still continue to experience joint symptoms that significantly impact their quality of life, a total knee arthroplasty (TKA) is advised [3]. Despite the high TKA success rate, approximately 20% of patients experience chronic post-TKA pain [4–6]. Various biopsychosocial contributors have shown to be associated with this chronic post-TKA pain [7].

One notable potential biological contributor to chronic post-TKA pain is hypersensitivity of the central nervous system [7–10]. This is reflected in the disturbance of somatosensory functioning, leading to hyperexcitability of the facilitatory ascending nerve pathways, along with reduced descending inhibition and changes in brain structures [11, 12]. Quantitative sensory testing (QST) and the Central Sensitization Inventory (CSI) are often used to measure this central nervous system disturbance[13], and disturbed somatosensory processing itself has been reported to be associated with chronic post-TKA pain [7, 9]. KOA pain is currently categorized as “chronic secondary MSK pain,” which means that pain is associated and maintained by the osteoarthritis disease itself [14]. Interestingly, one might expect that if all KOA patients suffered “chronic secondary MSK pain” solely [14], the pain and possible disturbed somatosensory functioning would resolve after effective treatment of KOA (i.e., TKA). This would imply that the disturbed somatosensory functioning is more peripherally driven (i.e., caused by an ongoing source of nociception and therefore indeed “chronic secondary MSK pain”). However, as ± 20% of patients continue experiencing chronic pain after TKA, and considering that the normalization of somatosensory functioning is not consistent in KOA patients after TKA [4, 6, 12, 15], this theory is being challenged.

Hence, it is postulated that in a subgroup of KOA patients, pain and somatosensory disturbances are more centrally driven, less reliant on peripheral source of nociception (and rather to be classified as “chronic primary MSK pain,” in which pain has become a condition on its own right [14]). Consequently, this subgroup may not experience full amelioration of pain and disturbed somatosensory functioning after surgery. This finding would warrant a broader treatment approach beyond the exclusive focus on the peripheral aspect, such as a more comprehensive modern neuroscience approach, including pain neuroscience education, cognitive behavioral therapy, and cognition-targeted exercise therapy [16, 17].

In light of these considerations, a previous systematic review showed that unfortunately most studies lacked subgrouping based on somatosensory functioning in KOA patients undergoing TKA, despite the association between improvement in some somatosensory functioning parameters and a pain improvement over time [15]. Two studies in the UK compared KOA patients undergoing TKA based on somatosensory functioning preoperatively, finding higher postoperative pain scores 6 months post-TKA [18], or a higher proportion of patients with moderate to severe 1 year post-TKA pain [19] in a neuropathic-like pain group compared to a nociceptive pain group. However, their somatosensory functioning categorization was limited to only preoperative neuropathic pain–like symptoms using the painDETECT questionnaire [18–20]. Two other studies in Denmark used somatosensory functioning as outcome variable and compared chronic postoperative pain groups (one after TKA [21] and one after total hip arthroplasty [22]), but only found between-group differences regarding temporal summation. However, none of the previous studies explored differences in post-TKA pain scores or their evolution over time between different somatosensory functioning evolution groups. This approach has the potential to improve our current understanding of pain mechanisms in KOA and post-TKA, as well as to identify possible subgroups of KOA patients.

Consequently, this study aimed to determine whether the change in pain intensity over time and pain intensity scores after TKA differed between somatosensory functioning evolution profiles in KOA patients undergoing TKA. Therefore, three somatosensory evolution profiles were defined and patients were classified accordingly. The hypothesis was that patients who experienced normal somatosensory functioning before and after TKA surgery (i.e., normal somatosensory functioning group or no indices for central sensitization) and patients who experienced disturbed somatosensory functioning before TKA surgery, but normalized somatosensory functioning after TKA (i.e., recovered somatosensory functioning group as an index for peripherally driven central sensitization) had more pain improvement or better pain scores after TKA compared to patients who experienced disturbed somatosensory functioning before and after TKA (i.e., persistent disturbed somatosensory functioning group as index for centrally driven central sensitization).

## Materials and methods

The Strengthening The Reporting of Observational studies in Epidemiology (STROBE) guidelines for cohort studies were used to conduct this multi-center longitudinal prospective cohort study [23]. The protocol is registered at clinicaltrials.gov (NCT05380648).

### Setting and participants

KOA patients awaiting TKA were recruited in the University Hospital of Antwerp and AZ Monica in Belgium, and the academic Hospital of Maastricht and St. Jans Gasthuis Weert in the Netherlands between March 2018 and July 2022. The study was approved by the respective ethical committees (BE300201319366 and NL6465408618).

Participants were eligible if diagnosed with KOA, were awaiting TKA, and aged ≥ 40 years. They were excluded if they experienced neurological or systemic diseases possibly impacting their pain, and were unable to speak or understand Dutch. After signing informed consent, participants completed a demographic, a somatosensory functioning (grouping variable), and a pain-related questionnaire (outcome variable) on paper or online via Qualtrics (www.qualtrics.com). After a practical skills training, two executive researchers (S.V. or L.M.) conducted the QST measurements (other grouping variables) at the Sensoric Functioning Lab (M2SENS) at the University of Antwerp’s campus “Drie Eiken” (Belgian participants), or at the orthopedic department of the academic Hospital of Maastricht and St. Jans Gasthuis Weert (Dutch participants) with standardized measurement forms. As this was a longitudinal study, data collection occurred between March 2018 and July 2023 at the following time points: 4 weeks pre-TKA (baseline), 3 months, and 1 year post-TKA. All participants had to stop first-stage pain medication, coffee, and alcohol 24 h before the physical measurements.

### Outcome variable

The outcome variable “pain intensity evolution from baseline to 3 months and 1 year post-TKA” was measured with the Knee Injury and Osteoarthritis Outcome Score (KOOS) subscale pain. The questionnaire comprises nine questions with a percentage score ranging from 0 (worst pain) to 100 (no pain) [24]. The KOOS is a reliable and valid questionnaire in KOA patients [25, 26].

### Group classifications

Indices of somatosensory functioning were assessed at baseline and 1 year post-TKA with the Central Sensitization Inventory (CSI) and QST. The CSI, pressure pain thresholds [PPTs], heat allodynia, temporal summation [TS], and conditioned pain modulation [CPM] were used to make group classifications. More details about the measurement methods [27–30] and the decision about “normal” somatosensory functioning [31–36] can be found in Table 1.

**Table 1:** Measurement methods and interpretation for normal somatosensory functioning. Abbreviations: *NRS* numeric rating scale, *PPT* pressure pain threshold, *CSI* Central Sensitization Inventory, *CPM* conditioned pain modulation

	Measurement method and device	Localization and position of participant	Normal somatosensory functioning
PPT	Method: A probe (1 cm^2^) was placed perpendicular to the test surface and pressure was increased until the subject, in supine position, reported a feeling of discomfort. An average of two measurements, separated by a pause of 30 s, was taken for analysis^25^ Device: Hand-held pressure algometer (*Wagner FDX 25 Force Gage*, *USA*)	Localization: Medial and lateral knee joint-line of the affected knee **(local PPT – combined)**, the m. ECRL of the non-dominant side, and the forehead **(widespread PPTs—combined)** Position: Supine	Despite the absence of clear cut-off or normative values for PPTs ^28^, patients were categorized using median scores, where higher scores indicated normal somatosensory functioning (post hoc determined)
Heat allodynia	Method: A roll-movement was performed for 10 s, after which participants scored their pain intensity on a NRS ranging from zero (no pain) to 10 (unbearable pain) Device: Thermal rollers (Rolltemp II Somedic Senselab) at 40 °C (hot stimulus)	Localization: Medial and lateral knee joint-line of the affected knee **(local heat allodynia – combined)**, and the m. ECRL of the non-dominant side **(widespread heat allodynia)** Position: Supine	No pain (< 1/10 on NRS) was interpreted as normal somatosensory functioning^29^ (a priori determined)
Temporal summation	Method: Thirty pinpricks were given at a pace of 1 pinprick/second, of which the first and last pinprick were scored for pain intensity on NRS ranging from zero (no pain) to 10 (unbearable pain). The differences between the NRS scores of the first and last pinprick were calculated and used for analysis^26^ Device: Von Frey monofilament of 60 g	Localization: Medial knee joint-line and wrist of the affected side **(combined to one variable)** Position: Supine	A difference of < two points on NRS score difference (last—first stimulus) was interpreted as normal somatosensory functioning^30,31^ (a priori determined)
CSI	A questionnaire with 25 questions, rates self-reported sensitization-associated symptoms (somatic and emotional symptoms, as well as pain sensitivity–related questions such as morning fatigue, anxiety attacks, poor sleep, light sensitivity, teeth grinding,.. etc.) on a five-point Likert scale^23,24^	/	A score ≥ 40 indicates the presence of self-reported symptoms of central sensitization^27^ (a priori determined)
CPM	Method: First, a temperature corresponding to a pain intensity NRS score of 4/10 (up to a maximum of 46 °C) was identified. This identified temperature (or 46 °C when the 4/10 on a NRS was not reached) was used as test stimulus. The participant had to score the test stimulus on a NRS 4 times. After a pause of 120 s, a conditioning stimulus (with a temperature of 0.5 °C more than the test stimulus) was added for 65 s and 20 s after its initiation, the test stimulus was repeated. Again, the participants had to score their pain for 4 times, but only on the test site. If the NRS at 46 °C and the mean of the NRS of test stimulus was equal to zero, the participant was excluded for analysis. The relative CPM scores ((absolute score [NRS score during conditioning stimulus – NRS score during only test stimulus]/NRS score during test stimulus) * 100) were used for analysis^26^ Device: Q-sense CPM device (Medoc, USA)	Localization: Test stimulus: the wrist of the affected side Conditioning stimulus: the wrist of the non-affected side Position: Supine	CPM values were categorized as patients being a facilitator (positive CPM values, indicating a less-efficient CPM), a non-responder (value 0, indicating no CPM response), and an inhibitor (negative CPM values, indication a more efficient CPM) ^32^ (a priori determined)

For each somatosensory functioning variable (local PPT, widespread PPT, local heat allodynia, widespread heat allodynia, TS, CPM, and CSI) criteria were defined to categorize participants as “normal somatosensory functioning,” “recovered somatosensory functioning,” or “persistent disturbed somatosensory functioning.” This categorization was done for each single variable, and as such the number of participants in the somatosensory functioning groups differed slightly for each variable. Details about this categorization can be found in Table 2.

**Table 2:** Categorization of somatosensory functioning groups. Abbreviations: *NRS* numeric rating scale, *CPM* conditioned pain modulation

	Normal somatosensory functioning	Recovered somatosensory functioning	Persistent disturbed somatosensory functioning
Pressure pain threshold	A score of above the group median at baseline and follow-up	A score below the group median at baseline, but above the group median at follow-up	A score below the group median at baseline and follow-up
Heat allodynia	A score < 1/10 on NRS (no pain)	A score of ≥ 1/10 on NRS at baseline, but < 1/10 on NRS (no pain) at follow-up	A score of ≥ 1/10 on NRS at baseline and follow-up
Temporal summation	An NRS difference score (last—first stimulus) of < two points	An NRS difference score (last—first stimulus) of < two points at baseline, but a difference of ≥ two points at follow-up	An NRS difference score (last—first stimulus) of ≥ two points at baseline and follow-up
Central sensitization inventory	A score of < 40	A score of ≥ 40 at baseline, but a score of < 40 at follow-up	A score of ≥ 40 at baseline and follow-up
Conditioned pain modulation	Being an inhibitor (negative CPM values, indicating a more efficient CPM)	Being a facilitator (positive CPM values, indicating a less-efficient CPM) or non-responder (a score of 0, no CPM response) at baseline, but inhibitor at follow-up	Being a facilitator (positive CPM values, indicating a less-efficient CPM) or non-responder (a score of 0, no CPM response) at baseline and follow-up

### Sample size

The sample size calculation of this project was based on the method of Diggle et al. [37]. Considering a minimal clinically important difference (MCID) of eight points in the KOOS subscale pain, 16 points as within-group standard deviation after TKA [24, 38], three measurement points, a confidence level of 0.05, and power of 0.80, at least 25 subjects per group were necessary [37]. Anticipating disturbed somatosensory functioning in 30% of KOA patients [10, 39], we hypothesized that 15% would have disturbed somatosensory functioning at baseline and 1 year post-TKA. Therefore, at least 223 participants were necessary to recruit to encounter a loss-to-follow-up of 25%.

### Statistical analyses

Statistical analyses were conducted using the IBM Statistical Package for Social Sciences Version 29 (SPSS, IBM Corporation, Armonk, NY) and R software (version 4.2.3) for multiple imputation. Boxplots were used to check univariate outliers, which were only deleted if unreasonable. Missing data were handled with multiple imputation (*n* = 10 imputed datasets) using predictive mean matching with the “mice” package in R [40]. To decrease the amount of grouping variables for defining somatosensory functioning groups, univariate association analyses using the Pearson correlation and Wilcoxon rank-sum tests between the different QST variables were performed. When variables were at least moderately correlated (correlation coefficient *r* ≥ 0.40), they were merged by taking the average of both values (if they measured the same somatosensory construct), and otherwise, only one variable was chosen for further analyses based on expertise and consistency with previous research. Demographic data was presented as mean and standard deviation (continuous data), and as number and frequency (categorical data). All data was pooled according to Rubin’s rules [41].

Thereafter, seven linear mixed models for repeated measures analyses were performed (local and widespread PPT and heat allodynia, TS, CPM, and CSI used to make seven normal, resolved, and persistent disturbed somatosensory functioning groups). Time, somatosensory functioning group, time x somatosensory functioning group (interaction term), and covariates (age and sex) were used as fixed effects. Subject identification was used as random effect. Residuals were checked for normality with a histogram and homogeneity of variance with a scatterplot. The median *p*-value of the interaction of all imputed datasets was calculated [42]. Least squares estimated means intervals and 95% confidence intervals were calculated and pooled according to Rubin’s rules [41]. Within-group, between-group at each time point, and interaction results are reported. A Benjamini–Hochberg correction was applied to correct for multiple testing and the significance level was therefore set to *p* < 0.028 [43]. If results were significant, post hoc analyses were performed, and a Bonferroni correction was applied to the post hoc *p*-values and corrected to *p* < 0.05.

## Results

### Participants

The study included 223 KOA participants with a mean age of 66 years old (standard deviation [SD] = 7.66) and 111 (49.8%) being female. Most participants had TKA surgery in AZ Monica (129 or 58% of participants), followed by SJG Weert (51 or 23% of participants, University Hospital of Antwerp (41 or 18% of participants), and University Hospital of Maastricht (2 or 1% of participants). Out of the 223 participants, 166 (75% of participants) had a Kellgren and Lawrence scale 3 or 4 (the higher, the worse structural KOA). Eighteen participants (8% of participants) were tested > 4 weeks preoperatively due to COVID-19 surgery postponement; however, no differences between groups regarding outcome variable and group division were found (*p* > 0.05).

### Missing data analysis

The KOOS subscale pain had 5.4% (12 participants) missing data at baseline, 22.0% (49 participants) at 3 months post-TKA, and 24.7% (55 participants) at post-TKA. Baseline missingness was mainly due to participants who forgot to complete questionnaires before surgery, while missingness at follow-up was due to exclusion of participants (diagnosed with rheumatoid arthritis diagnosis, cancer, or neuropathic pain symptoms in the lower legs due to hernia – 2.3%, 5 participants), and primarily from losses-to-follow-up (unreachable, time constraints, or planned revision – 22.4%, 50 participants). Grouping variables had missing data ranging from 1.3 to 34.1% (3 to 76 participants). The missing data at baseline stemmed from participants absent during the planned physical testing (1.3%, 3 participants), absence of the baseline PPT measured at forehead because of protocol updates at February 2019 for future project purposes (17%, 38 participants), and missing CPM data due to device issues or reported absence of pain during test stimulus (10.8%, 22 participants). At follow-up, missing data was due to the same reasons as missingness in the KOOS subscale pain.

Details can be found in Supplementary table S1. Because multiple imputation handled missing data, all participants (*n* = 223) were analyzed.

### Group division

To avoid an overload of group classifications and to manage the interpretation of the somatosensory functioning groups correlated QST variables of the same construct were combined and averaged: (a) PPTs measured at medial and knee joint-line were merged into one local PPT (*r* = 0.711–0.764), (b) PPTs measured at m. Extensor carpi radialis longus and the forehead were merged into one widespread PPT (*r* = 0.650–0.721), (c) heat allodynia measured at medial and lateral knee joint-line was bundled into local heat allodynia (*r* = 0.640–0.702), and (d) TS measured at the medial knee joint-line and medial wrist was also bundled into TS in general (*r* = 0.418–0.501). Regional PPT (measured at m. Tibialis anterior) and cold allodynia were not reported as grouping variables, because of their moderate to high correlation with local (*r* = 0.686–0.805) and widespread PPT variables (*r* = 0.526–0.726), and heat allodynia variables (*r* = 0.561–0.727), respectively (supplementary table S2).

Regarding the separate somatosensory functioning groups, the number of participants varied depending on QST variables or CSI used for subgrouping: 15.07 to 77.13% (34 to 172 participants) for normal somatosensory functioning, 9.87 to 22.42% (22 to 50 participants) for recovered somatosensory functioning, and 12.11 to 62.33% (27 to 139 participants) for persistent disturbed somatosensory functioning (Table 3).

**Table 3:** Number and percentage of participants divided by somatosensory functioning group. Abbreviations: *PPT* pressure pain threshold, *THA* thermal heat allodynia, *TS* temporal summation, *CPM* conditioned pain modulation, *CSI* Central Sensitization Inventory

*N* (% of total sample)	Normal somatosensory functioning	Recovered somatosensory functioning	Persistent disturbed somatosensory functioning
Local PPT	83 (37.22)	40 (17.94)	100 (44.84)
Widespread PPT	81 (36.32)	43 (19.28)	99 (44.39)
Local THA	142 (63.68)	30 (13.45)	51 (22.87)
Widespread THA	133 (59.64)	22 (9.87)	68 (30.49)
TS	132 (59.19)	44 (19.73)	47 (21.08)
CSI	172 (77.13)	24 (10.76)	27 (12.11)
CPM	34 (15.07)	50 (22.42)	139 (62.33)

### Results of change in pain intensity after surgery in different somatosensory evolution groups

**Fig. 1:**
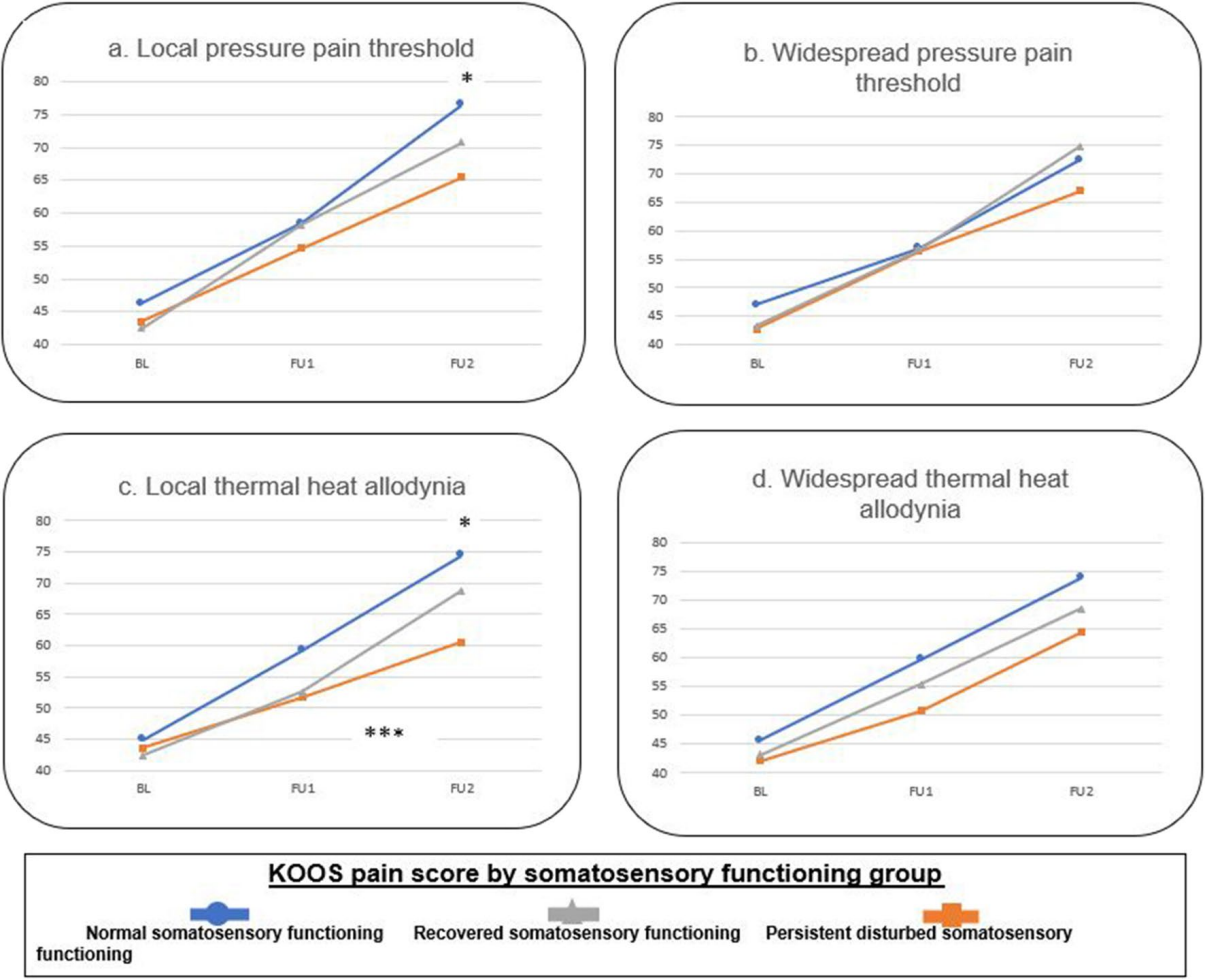
Evolution of Knee Injury and Osteoarthritis Outcome Score subscale pain over time in the different somatosensory functioning groups for pressure pain threshold and thermal allodynia. * = significant different between normal and persistent disturbed somatosensory group at 1 year postoperative. ** = significant different between normal and recovered somatosensory functioning group at baseline. *** = significant interaction effect (time*group)

**Fig. 2:**
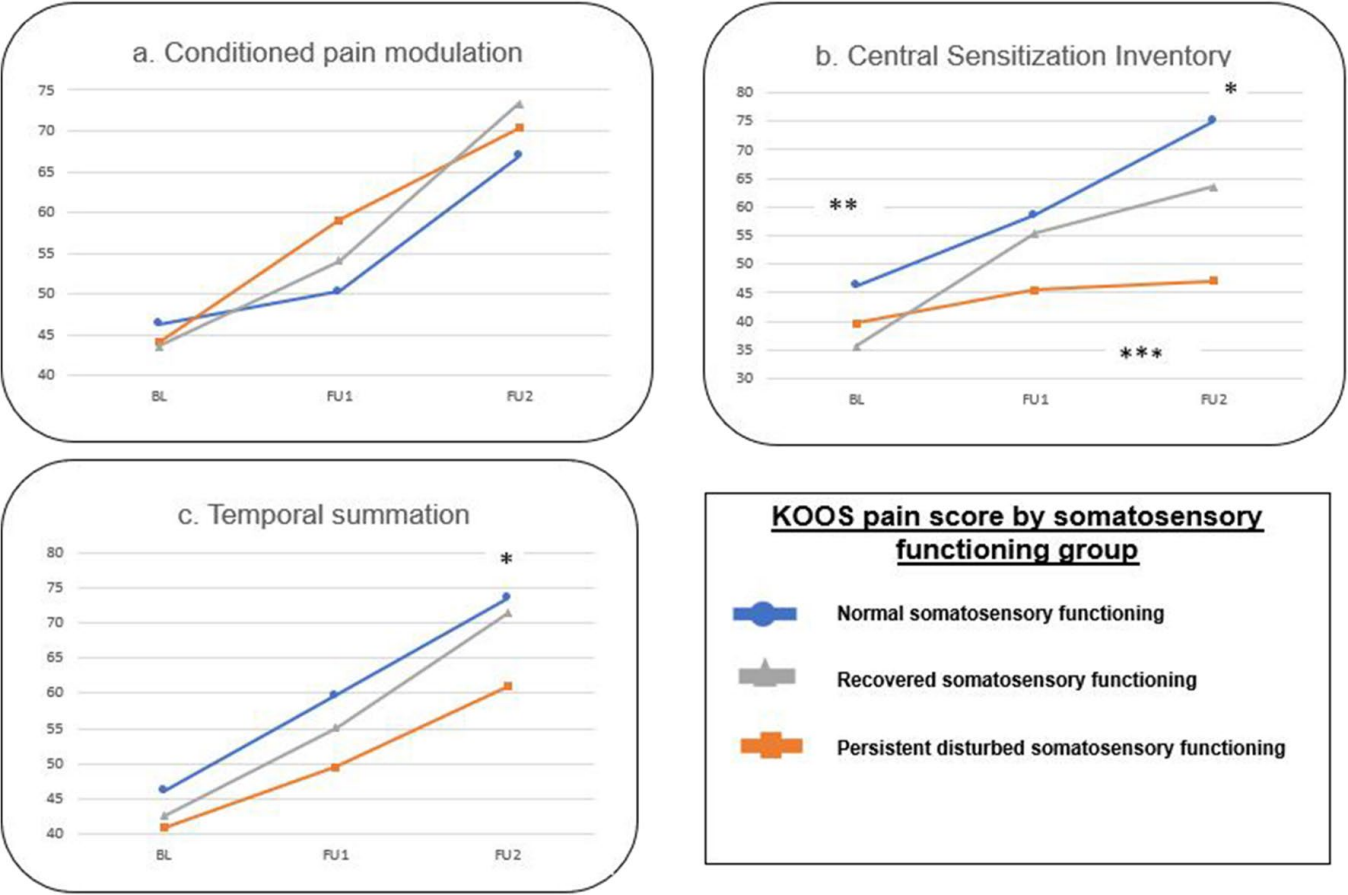
Evolution of Knee Injury and Osteoarthritis Outcome Score subscale pain over time in the different somatosensory functioning groups for temporal summation, conditioned pain modulation, and the Central Sensitization Inventory. * = significant different between normal and persistent disturbed somatosensory group at 1 year postoperative. ** = significant different between normal and recovered somatosensory functioning group at baseline. *** = significant interaction effect (time*group)

Detailed results can be found in Figs. 1 and 2 and Table 4.

**Table 4:** Evolution of Knee Injury and Osteoarthritis Outcome Score subscale pain over time in the different somatosensory functioning groups. All *p*-values (within-group, between-group at each time point, and interaction term) < 0.028* (Benjamini–Hochberg correction), all post hoc *p*-values underwent a Bonferroni correction and p-value set to < 0.05*, all no reported post hoc *p*-values > 0.05. Abbreviations: *BL* = baseline, *FU* = follow-up, *CI* = confidence interval, *KOOS* = Knee Injury and Osteoarthritis Outcome Score, *PPT* = pressure pain threshold, *TA* = m. Tibialis anterior, *MK* = medial knee, *LK* = lateral knee, *ECRL* = m. Extensor carpi radialis longus, *FH* = forehead, *TCA* = thermal cold allodynia, *THA* = thermal heat allodynia, TS = temporal summation, CPM = conditioned pain modulation, CSI = Central Sensitization Inventory

Grouping variable	Time point	Normal somatosensory functioning	Persistent disturbed somatosensory functioning	Recovered somatosensory functioning	*p*-value between groups at each time point and interaction (time*group)	*p*-value post hoc between groups at each time point and interaction
Estimated mean (95% CI) of KOOS subscale pain						
Local PPT	BL	46.24 (41.03, 51.45)	43.50 (38.56, 48.44)	42.53 (34.72, 50.33)	0.306	
	FU1	58.45 (52.25, 64.66)	54.64 (48.78, 60.50)	58.20 (50.12, 66.28)	0.618	
	FU2	76.53 (70.54, 82.52)	65.50 (59.95, 71.05)	70.82 (59.92, 81.72)	0.009*	Normal vs. persistent: 0.009*
*p*-value time effect within-group		< 0.001*	< 0.001*	< 0.001*	Time*group: 0.202	
Widespread PPT	BL	46.96 (41.50, 52.42)	42.68 (37.43, 47.94)	43.22 (35.91, 50.54)	0.080	
	FU1	56.95 (51.11, 62.80)	56.42 (50.93, 61.91)	56.71 (49.04, 64.39)	0.862	
	FU2	72.51 (67.07, 77.94)	67.03 (61.01, 73.06)	74.84 (65.68, 84.00)	0.238	
*p*-value time effect within-group		< 0.001*	< 0.001*	< 0.001*	Time*group: 0.498	
Local THA	BL	44.99 (41.13, 48.85)	43.61 (36.74, 50.48)	42.38 (33.96, 50.81)	0.612	
	FU1	59.30 (55.18, 63.42)	51.68 (43.41, 59.95)	52.60 (40.63, 64.58)	0.221	
	FU2	74.46 (70.51, 78.42)	60.52 (50.18, 70.86)	68.78 (58.31, 78.64)	0.003*	Normal vs. persistent: 0.003*
*p*-value time effect within-group		< 0.001*	< 0.001*	< 0.001*	Time*group: 0.027*	Normal vs persistent BL to FU2: 0.018*
Widespread THA	BL	45.62 (41.63, 49.60)	42.12 (36.05, 48.18)	43.23 (33.02, 53.45)	0.359	
	FU1	59.76 (55.64, 63.88)	50.82 (43.17, 58.47)	55.43 (42.78, 58.09)	0.084	
	FU2	73.87 (69.50, 78.23)	64.44 (56.34, 72.54)	68.46 (56.89, 80.03)	0.066	
*p*-value time effect within-group		< 0.001*	< 0.001*	< 0.001*	Time*group: 0.519	
Temporal summation	BL	46.11 (42.00, 50.23)	40.91 (32.07, 49.75)	42.60 (35.49, 49.71)	0.058	
	FU1	59.69 (55.46, 63.92)	49.55 (39.60, 59.51)	55.06 (47.87, 62.25)	0.067	
	FU2	73.55 (69.17, 77.93)	60.92 (51.67, 70.17)	71.42 (63.96, 78.89)	0.027*	Normal vs. persistent: 0.027*
*p*-value time effect within-group		< 0.001*	< 0.001*	< 0.001*	Time*group: 0.344	
CPM	BL	46.33 (38.03, 54.64)	44.13 (40.04, 48.21)	43.57 (36.63, 50.50)	0.516	
	FU1	50.33 (40.69, 59.98)	59.10 (54.46, 63.74)	54.10 (44.76, 63.44)	0.156	
	FU2	66.85 (57.29, 76.42)	70.40 (65.82, 74.97)	73.27 (65.65, 80.90)	0.377	
*p*-value time effect within-group		< 0.001*	< 0.001*	< 0.001*	Time*group: 0.100	
CSI	BL	46.26 (42.83, 49.68)	39.73 (30.80, 48.65)	35.65 (25.42, 45.87)	0.003*	Normal vs. recovered: 0.010*
	FU1	58.58 (54.87, 62.30)	45.44 (34.90, 55.98)	55.34 (43.24, 67.44)	0.106	
	FU2	75.07 (70.88, 79.25)	47.08 (35.53, 58.64)	63.48 (53.29, 73.68)	< 0.001*	Normal vs. persistent: < 0.001*, recovered vs. persistent: 0.044*
*p*-value time effect within-group		< 0.001*	0.213	< 0.001*	Time*group: 0.003*	Normal vs. persistent BL to FU2: 0.001*

## Interaction effect (time*group)

Only differences in changes of the KOOS subscale pain over time were found between the normal, resolved, and persistent disturbed somatosensory functioning groups classified according to local heat allodynia (*p* = 0.011) and CSI (*p* < 0.001). No differences were found regarding the other somatosensory functioning grouping variables (*p* > 0.028). Regarding these two significant grouping variables, post hoc analyses showed that the persistent disturbed somatosensory group had less pain improvement from baseline to 1 year post-TKA compared to the normal somatosensory functioning group (*p* = 0.018 and *p* = 0.001, respectively). Other post hoc analyses were non-significant (*p* > 0.05).

## Within-group time effect

All somatosensory functioning groups classified according to the seven grouping variables experienced an improvement of the KOOS subscale pain score from baseline to 3 months and 1 year after the TKA (*p* < 0.001), except for the persistent disturbed somatosensory group classified according to the CSI, which showed no improvement over time (*p* = 0.213).

## Between-group effect at each time point

Differences between somatosensory functioning groups classified according to local PPT (*p* = 0.009) and heat allodynia (*p* = 0.003), temporal summation (*p* = 0.027), and CSI (*p* < 0.001) were found at 1 year post-TKA. At baseline, also differences between groups classified according to CSI were found (*p* = 0.003). At 1 year post-TKA, post hoc analyses showed that the persistent disturbed somatosensory functioning group had worse pain scores compared to the normal somatosensory group (*p* = 0.009 for local PPT, *p* = 0.003 for local heat allodynia, *p* = 0.027 for temporal summation, and *p* < 0.001 for CSI), and compared to the recovered somatosensory group (*p* = 0.044 for CSI). At baseline, the recovered somatosensory functioning group had worse pain scores compared to the normal somatosensory functioning group (*p* = 0.003 for CSI). No other post hoc differences could be found (*p* > 0.05).

## Discussion

This study aimed to determine whether the change in pain intensity over time differs between somatosensory functioning evolution profiles in KOA patients undergoing TKA. This study revealed that the three somatosensory functioning subgroups (separately classified according to all seven grouping variables) decreased in pain score (= less pain) from baseline to 3 months and 1 year post-TKA, except for the persistent disturbed somatosensory group classified according to the CSI which had no change in pain score over time. In addition, the persistent disturbed somatosensory functioning group had less pain improvement from baseline to 1 year post-TKA, and worse pain intensity scores at 1 year post-TKA compared to the normal somatosensory group classified according to local heat allodynia and CSI. Moreover, the same subgroup classified according to the CSI also exhibited worse pain intensity scores at 1 year post-TKA compared to the recovered somatosensory functioning group. The persistent disturbed somatosensory functioning group classified according to local PPT and TS also presented worse pain intensity scores 1 year post-TKA compared to the normal somatosensory functioning group.

### Interpretation of findings

Our hypothesis of no or less pain improvement or worse pain scores 1 year post-TKA in the persistent disturbed somatosensory functioning group (i.e., indicative of centrally driven central sensitization) compared to the other groups was only confirmed with the difference in pain improvement over time or pain intensity 1 year post-TKA between the normal and persistent disturbed somatosensory group classified according to four of the seven grouping variables. This aligns with the notion that, especially in the persistent disturbed somatosensory functioning group, other factors can contribute to persistent post-TKA pain [44], beyond the peripheral source of nociception (KOA), and are often overlooked factors in current rehabilitation [45, 46].

No differences between the recovered somatosensory functioning group and the other groups were found, except for the 1 year post-TKA pain score between the recovered and persistent disturbed somatosensory functioning groups according to the CSI group classification. The absence of differences in the QST grouping classification variables could suggest the likelihood that chronic post-TKA pain is also associated with various other preoperative variables (including also psychological, sociodemographic, and functional factors [7]), beyond specific somatosensory dysfunction. This plausible theory gains support from the highly clinically relevant differences in the CSI grouping variable, which also includes questions about state psychological factors (a dimension not covered by QST). It is possible that delving more into the evolution of psychological variables, commonly associated with primary chronic pain [14] and not limited to somatosensory dysfunction, may reveal additional distinctions. However, future research should confirm or refute this proposition.

Notably, pain intensity values at 1 year post-TKA of the recovered somatosensory functioning group are in between the values of the other two groups. Better scores were seen compared to the persistent disturbed somatosensory functioning group, but worse compared to the normal somatosensory functioning group (except for groups based on CPM or widespread PPT). This might be an indication that chronic pain indeed needs to be approached as a continuum, meaning that overlap between different mechanisms (e.g., no, peripherally, or centrally driven disturbed somatosensory functioning in the current study) can be present [47].

Another possible explanation for the absence in differences between the recovered and persistent disturbed somatosensory functioning group is, apart from the cut-off of 40 on the CSI [31], a consensus about the optimal methodology to assess disturbed somatosensory functioning, including normative and cut-off values is lacking. While we adhered to previous literature and theoretical rationale [33–36] in defining persisted disturbed vs. non-disturbed somatosensory functioning groups using QST methods, it should be acknowledged that this is an exploratory effort, emphasizing the need for confirmation in future research.

### Relation to previous literature

Two previous studies on somatosensory functioning subgroups in KOA patients undergoing TKA [18] showed that the preoperative disturbed somatosensory functioning group had higher postoperative pain intensity scores 6 months post-TKA, or a higher proportion of participants with moderate-to-severe 1 year post-TKA pain [19] compared to the normal somatosensory functioning group. This aligns with four of our grouping variables, but contrasts with the other three. More specifically, our study revealed that this difference was only seen between the normal and persistent disturbed somatosensory functioning group, and not between the recovered and normal somatosensory group, suggesting that preoperative disturbed somatosensory functioning alone is not as strongly associated with worse post-TKA pain scores as pre- and postoperative disturbed somatosensory functioning. Importantly, these studies relied on baseline painDETECT scale scores to form subgroups (high neuropathic-like pain symptoms vs. low neuropathic-like pain symptoms), lacking focus on other specific somatosensory functioning variables and longitudinal changes as in the current study.

Two additional studies in osteoarthritis also adopted subgroup analyses instead of focusing on osteoarthritis patients in general, using chronic pain after surgery (NRS pain score at 12 months post-TKA ≥ 3 [21], or NRS pain score at 6 weeks post total hip arthroplasty > 0 [22]) or not (NRS pain score at 12 months < 3, or NRS pain score = 0) as grouping variable, and somatosensory functioning as outcome variables. Petersen et al. [21] showed significant improvement of all PPTs after surgery in the no chronic pain group, while the chronic pain group only had significant improvement for widespread PPT. However, no between-group differences were significant. Similarly, Izumi et al. [22] found no differences regarding PPT outcomes. The current study found between-group differences classified according to local PPT for 1 year post-TKA pain, which is in contrast to Petersen et al. [21], but no differences between-groups classified according to widespread PPT, aligning with both studies [21, 22]. Concerning TS, within-group analyses in Izumi et al. [22] revealed improvement in the no pain group after surgery, but not in the pain group. In addition, Petersen et al. [21] also showed worse TS values in the chronic pain subgroup compared to the no chronic pain group at 12 months post-TKA. The current study found that all subgroups classified according to TS improved in pain intensity over time, but between-group differences classified according to TS were also found at 1 year post-TKA. No differences for CPM were found in both studies [21, 22], which is also in line with findings of the current study.

### Implications for future research and clinical practice

The present study represents an initial effort in subgrouping based on somatosensory profiles. However, future research should further validate these variables and methods to accurately capture somatosensory functioning groups in KOA patients due to the existing variability in QST methods [48], including cut-offs and normative values. In clinical practice, recognizing the potential existence of a “centrally driven central sensitization” subgroup in KOA patients, as indicated by the presence of self-reported central sensitization according to baseline and 1 year post-TKA CSI scores in the current study, can be relevant. Healthcare professionals may consider additional therapeutical approaches for this subgroup, such as multidisciplinary pain management programs [49], next to the more peripheral focus of today to achieve comprehensive pain relief [16, 17]. This could additionally have positive influence on healthcare and society, as lower healthcare and society costs are expected when the disorder and source of pain are more adequately targeted [50, 51].

### Strengths and limitations of the study

This study presents with several strengths. First, this study has taken the first step to account for differences in somatosensory functioning evolution within the KOA population and whether this is related to the evolution of pain intensity over time. Next, thorough statistical analyses including appropriate missing data analysis in combination with the presentation of a broad spectrum of different somatosensory functioning grouping variables were performed. A limitation of this study is the broad range of sample sizes in the different somatosensory functioning groups. However, the amount of grouping variables was kept to a minimum by bundling local and widespread measurements. The different QST variables were presented separately, because they measure different constructs of (possible) disturbed somatosensory functioning (CPM measures the endogenous pain inhibition system, TS measures the excitability of the ascending pathways, etc.) [12]. However, studies that validate the ideal methods to assess somatosensory functioning, cut-offs, and normative values are necessary. Last, also the CPM method, for which patients who had a NRS score of 0/10 on the test stimulus were excluded, is a possible limitation. It is possible that the noxious stimulus was too low to provoke a CPM effect and resulted in unexpected results**.**

## Conclusion

The present study classified KOA patients undergoing TKA into three somatosensory functioning evolution groups (normal, persistent disturbed, and recovered) based on seven variables that were considered proxies of somatosensory functioning. The study compared pain intensity evolution from baseline to post-TKA and pain intensity at 1 year post-TKA between the groups and found differences between the three groups classified according to four out of seven grouping variables (local PPT and heat allodynia, TS, and CSI). The most important finding was that the persistent disturbed somatosensory functioning group had less pronounced pain improvement (based on CSI and local heat allodynia) and had worse pain scores 1 year post-TKA (based on CSI, local PPT and heat allodynia, and TS) compared to the to normal somatosensory functioning group. The persistent disturbed somatosensory functioning group had also worse pain scores 1 year post-TKA compared to the recovered group classified according to the CSI. These are preliminary results suggesting a “centrally driven central sensitization” subgroup in KOA patients awaiting TKA, comprising their less pain improvement and disturbed somatosensory functioning after TKA. Future research should further validate methods, cut-offs, and normative values to adequately assess somatosensory functioning, including studies with bigger sample sizes regarding the disturbed somatosensory functioning group.

### Supplementary Information

Below is the link to the electronic supplementary material.

Supplementary file1 (PDF 140 KB)

Supplementary file2 (PDF 166 KB)

## Data Availability

The datasets generated during and analyzed during the current study are available from the corresponding author on reasonable request.
