# Supplementary material for: Does pain intensity after total knee arthroplasty depend on somatosensory functioning in knee osteoarthritis patients? A prospective cohort study
Source: Clin Rheumatol. 2024 Apr 26;43(6):2047–59. doi: 10.1007/s10067-024-06976-7 (PMC11111543; doi:10.1007/s10067-024-06976-7)
Supplement: Supplementary file 1 — Supplementary file1 (PDF 140 KB) [file 10067_2024_6976_MOESM1_ESM.pdf]

<u>**Online Resource 1: supplementary tables**</u>

Supplementary table S1: missing value analysis

Variable	Number of participants	Mean	Standard Deviation	Missing (count)	Missing (percent)
**Age**	223	65,5247	7,65636	0	0
**BMI BL**	220	29,9925	5,24961	3	1,3
**Hospital**	223	/	/	0	0
**OA-grade**	214	/	/	9	4,0
**sex**	219	/	/	0	0
**PPT m. Tibialis anterior BL**	220	50,8935	24,81421	3	1,3
**PPT m. Tibialis anterior FU2**	173	55,2214	28,42383	50	22,4
**PPT medial knee BL**	220	42,8318	23,70894	3	1,3
**PPT medial knee FU2**	173	44,8347	22,91417	50	22,4
**PPT lateral knee BL**	220	47,8264	26,11936	3	1,3
**PPT lateral knee FU2**	173	51,4208	26,33968	50	22,4
**PPT m. ECRL BL**	220	37,7762	18,05692	3	1,3
**PPT m. ECRL FU2**	173	39,9081	16,89023	50	22,4
**PPT forehead BL**	185	30,1805	12,72836	38	17,0
**PPT forehead FU2**	164	31,8652	11,09273	59	26,5
**TS medial knee BL**	220	1,2341	2,01501	3	1,3
**TS medial knee FU2**	172	0,6860	1,57288	51	22,9
**TS medial wrist BL**	219	0,9840	1,55804	4	1,8
**TS medial wrist FU2**	172	0,6453	1,16821	51	22,9
**Cold allodynia medial knee BL**	219	0,3607	0,95903	4	1,8
**Cold allodynia medial knee FU2**	173	0,1734	0,63244	50	22,4
**Heat allodynia medial knee BL**	219	0,8219	1,45900	4	1,8
**Heat allodynia medial knee FU2**	173	0,5491	1,13822	50	22,4
**Cold allodynia lateral knee BL**	219	0,2694	0,91146	4	1,8
**Cold allodynia lateral knee FU1**	181	0,1243	0,42461	42	18,8
**Cold allodynia lateral knee FU2**	173	0,0809	0,34831	50	22,4
**Heat allodynia lateral knee BL**	219	0,3653	1,08530	4	1,8
**Heat allodynia lateral knee FU2**	173	0,1850	0,63832	50	22,4
**Cold allodynia m. ECRL BL**	219	0,1941	0,76327	4	1,8
**Cold allodynia m. ECRL FU2**	173	0,1503	0,58116	50	22,4
**Heat allodynia m. ECRL BL**	219	0,4475	1,10492	4	1,8
**Heat allodynia m. ECRL FU2**	173	0,3873	0,90558	50	22,4
**CPM relative score BL**	201	14,3139	65,20998	22	9,9
**CPM relative score FU2**	148	6,2707	54,49017	75	33,6
**CSI BL**	211	28,0569	13,14268	12	5,4
**CSI FU2**	168	23,3988	13,81232	55	24,7
**KOOS subscale pain BL**	211	44,0699	15,31292	12	5,4
**KOOS subscale pain FU1**	174	15,4540	9,35465	49	22,0
**KOOS subscale pain FU2**	168	73,4582	24,07833	55	24,7

Abbreviations: OA= osteoarthritis, BMI = body mass index, KOOS = Knee Osteoarthritis Outcome and Index Score, CSI= Central Sensitization Inventory, m. = musculus, ECRL = Extensor capri radialis longus, PPT = pressure pain threshold, TS = temporal summation, CPM = conditioned pain modulation, CRP= creatinine phosphate, BL= baseline, FU1= follow-up 1, FU2= follow-up 2

Supplementary Table S2: correlation coefficients between quantitative sensory testing variables at baseline

	PPT m. TA	PPT mk	PPT lk	PPT m. ECRL	PPT forehead	TS mk	TS mw	CPM	TH cold mk	TH heat mk	TH cold lk	TH heat lk	TH cold m. ECRL	TH heat m. ECRL
**PPT m. TA**	1	0,796	0,805	0,726	0,656	-0,35	-0,223	-0,066	-0,175	-0,189	-0,213	-0,237	-0,148	-0,091
**PPT mk**	0,796	1	0,764	0,652	0,597	-0,306	-0,171	-0,131	-0,239	-0,221	-0,209	-0,23	-0,152	-0,106
**PPT lk**	0,805	0,764	1	0,721	0,694	-0,313	-0,27	-0,017	-0,271	-0,276	-0,265	-0,299	-0,232	-0,169
**PPT m. ECRL**	0,726	0,652	0,721	1	0,728	-0,326	-0,291	-0,043	-0,205	-0,192	-0,254	-0,261	-0,214	-0,176
**PPT forehead**	0,656	0,597	0,694	0,728	1	-0,318	-0,287	-0,01	-0,277	-0,219	-0,311	-0,291	-0,293	-0,243
**TS mk**	-0,35	-0,306	-0,313	-0,326	-0,318	1	0,418	0,064	0,166	0,118	0,209	0,157	0,098	0,061
**TS mw**	-0,223	-0,171	-0,27	-0,291	-0,287	0,418	1	-0,017	0,103	0,119	0,188	0,15	0,12	0,074
**CPM**	-0,066	-0,131	-0,017	-0,043	-0,01	0,064	-0,017	1	-0,019	-0,066	-0,092	-0,051	-0,154	-0,156
**TH cold mk**	-0,175	-0,239	-0,271	-0,205	-0,277	0,166	0,103	-0,019	1	0,517	0,708	0,471	0,575	0,355
**TH heat mk**	-0,189	-0,221	-0,276	-0,192	-0,219	0,118	0,119	-0,066	0,517	1	0,395	0,702	0,391	0,66
**TH cold lk**	-0,213	-0,209	-0,265	-0,254	-0,311	0,209	0,188	-0,092	0,708	0,395	1	0,49	0,664	0,355
**TH heat lk**	-0,237	-0,23	-0,299	-0,261	-0,291	0,157	0,15	-0,051	0,471	0,702	0,49	1	0,411	0,623
**TH cold m. ECRL**	-0,148	-0,152	-0,232	-0,214	-0,293	0,098	0,12	-0,154	0,575	0,391	0,664	0,411	1	0,475
**TH heat m. ECRL**	-0,091	-0,106	-0,169	-0,176	-0,243	0,061	0,074	-0,156	0,355	0,66	0,355	0,623	0,475	1

Abbreviations: CPM = conditioned pain modulation, CSI = Central Sensitization Index, ECRL = m. Extensor Carpi Radialis Longus, lk = lateral knee, mk = medial knee, mw = medial wrist, m. = musculus, PPT = pressure pain threshold, TA = m. Tibialis Anterior, TH = thermal hypersensitivity, TS = temporal summation

Supplementary Table S3: correlation coefficients between quantitative sensory testing variables at one-year postoperative

	PPT m. TA	PPT mk	PPT lk	PPT m. ECRL	PPT forehead	TS mk	TS mw	CPM	TH cold mk	TH heat mk	TH cold lk	TH heat lk	TH cold m. ECRL	TH heat m. ECRL
**PPT m. TA**	1	0,734	0,686	0,586	0,526	-0,144	-0,175	0,075	-0,037	-0,027	-0,025	-0,014	0,015	-0,012
**PPT mk**	0,734	1	0,711	0,647	0,655	-0,233	-0,21	0,022	-0,122	-0,166	-0,05	-0,156	-0,086	-0,082
**PPT lk**	0,686	0,711	1	0,496	0,586	-0,168	-0,167	0,034	-0,047	-0,04	0,007	-0,045	-0,043	-0,091
**PPT m. ECRL**	0,586	0,647	0,496	1	0,65	-0,173	-0,209	0,069	-0,144	-0,16	-0,108	-0,143	-0,149	-0,108
**PPT forehead**	0,526	0,655	0,586	0,65	1	-0,249	-0,223	0,04	-0,148	-0,222	-0,118	-0,13	-0,176	-0,203
**TS mk**	-0,144	-0,233	-0,168	-0,173	-0,249	1	0,501	-0,007	0,148	0,149	0,148	0,203	0,208	0,199
**TS mw**	-0,175	-0,21	-0,167	-0,209	-0,223	0,501	1	-0,1	0,374	0,268	0,195	0,261	0,265	0,363
**CPM**	0,075	0,022	0,034	0,069	0,04	-0,007	-0,1	1	-0,03	-0,01	0,003	-0,041	-0,021	-0,069
**TH cold mk**	-0,037	-0,122	-0,047	-0,144	-0,148	0,148	0,374	-0,03	1	0,655	0,61	0,594	0,659	0,602
**TH heat mk**	-0,027	-0,166	-0,04	-0,16	-0,222	0,149	0,268	-0,01	0,655	1	0,438	0,64	0,631	0,725
**TH cold lk**	-0,025	-0,05	0,007	-0,108	-0,118	0,148	0,195	0,003	0,61	0,438	1	0,336	0,576	0,327
**TH heat lk**	-0,014	-0,156	-0,045	-0,143	-0,13	0,203	0,261	-0,041	0,594	0,64	0,336	1	0,538	0,557
**TH cold m. ECRL**	0,015	-0,086	-0,043	-0,149	-0,176	0,208	0,265	-0,021	0,659	0,631	0,576	0,538	1	0,652
**TH heat m. ECRL**	-0,012	-0,082	-0,091	-0,108	-0,203	0,199	0,363	-0,069	0,602	0,725	0,327	0,557	0,652	1

Abbreviations: CPM = conditioned pain modulation, CSI = Central Sensitization Index, ECRL = m. Extensor Carpi Radialis Longus, lk = lateral knee, mk = medial knee, mw = medial wrist, m. = musculus, PPT = pressure pain threshold, TA = m. Tibialis Anterior, TH = thermal hypersensitivity, TS = temporal summation
